# CT-Derived Aortic Valve Anatomy and Acute Complications After Self-Expanding and Balloon-Expandable TAVI

**DOI:** 10.3390/medicina61091650

**Published:** 2025-09-11

**Authors:** Alexandru Antoniu Stan, Ayman Elkahlout, Marius Mihai Harpa, Marian Pop, Mihaly Veres, Antonela Delia Stan, Paul-Adrian Călburean, David Emanuel Aniței, Anda-Cristina Scurtu, Klara Brînzaniuc, Horațiu Suciu

**Affiliations:** 1 Doctoral School, George Emil Palade University of Medicine, Pharmacy, Science and Technology of Târgu Mureș, 540142 Târgu Mureș, Romania; 2 Department of Interventional Cardiology, Emergency Institute for Cardiovascular Diseases and Transplantation, 540136 Târgu Mureș, Romania; 3 Department of Surgery IV, George Emil Palade University of Medicine, Pharmacy, Science and Technology of Târgu Mureș, 540142 Târgu Mureș, Romania; 4 Department of Cardiovascular Surgery, Emergency Institute for Cardiovascular Diseases and Transplantation, 540136 Târgu Mureș, Romania; 5 Department of Radiology, George Emil Palade University of Medicine, Pharmacy, Science and Technology of Târgu Mureș, 540142 Târgu Mureș, Romania; 6 Department of Intensive Care, Emergency Institute for Cardiovascular Diseases and Transplantation, 540136 Târgu Mureș, Romania; 7 Faculty of Law, University of Bucharest, 030018 Bucharest, Romania; 8 Department of Biostatistics and Medical Informatics, George Emil Palade University of Medicine, Pharmacy, Science and Technology of Târgu Mureș, 540142 Târgu Mureș, Romania; 9 Department of Anatomy, George Emil Palade University of Medicine, Pharmacy, Science and Technology of Târgu Mureș, 540142 Târgu Mureș, Romania

**Keywords:** severe aortic stenosis, transcatheter aortic valve implantation, balloon-expandable valve, self-expandable valve, acute complications

## Abstract

*Background and Objectives*: This study aimed to assess the clinical and anatomical predictors of acute cardiac complications after transcatheter aortic valve implantation (TAVI). *Materials and Methods*: All patients who underwent a TAVI procedure for severe aortic stenosis between November 2016 and May 2025 at a tertiary center in Romania were screened for inclusion. Of those, patients who had available computer tomography valvular sizing reports were included in the present study. *Results*: A total of 485 patients were included in this study. Balloon-expandable valves were implanted in 381 patients (78.5%), while self-expanding valves were used in 104 patients (21.4%). A total of sixty-nine (14.2%) patients suffered at least one acute cardiac complication following TAVI, and in-hospital death occurred in nine (1.8%) patients. In the multivariable analysis, clinical parameters—such as diabetes mellitus, left bundle branch block, or left ventricular diameter—and anatomic parameters, such as left coronary artery height and sinotubular junction height, were predictors of acute complications. Similarly, periprocedural characteristics, such as maximum transprosthetic gradient and the use of the Portico/Navitor valve platform was also associated with the occurrence of acute complications. *Conclusions*: A high acute complications rate is typical for TAVI, although most complications can be successfully treated and the in-hospital death rate is low. Left coronary artery height and sinotubular junction height were predictors of acute complications, among other clinical and procedural characteristics.

## 1. Introduction

Transcatheter aortic valve implantation (TAVI) has transformed the management of severe aortic stenosis, offering a less invasive alternative to surgery for an expanding range of patients [1,2]. However, acute procedural complications remain a significant concern, as they can offset the benefits of TAVI and impact both short- and long-term outcomes. Contemporary studies and trials indicate that certain complications, notably conduction disturbances, paravalvular regurgitation, and vascular injuries, occur more frequently with TAVI than with surgical aortic valve replacement (SAVR) [3]. As TAVI is now being used in younger, lower-risk patients with longer life expectancies, there is a pressing need to minimize these complications [4]. This has driven extensive research into the incidence, impact, and predictors of acute complications following TAVI.

The most common acute complications after TAVI include new conduction disturbance leading to high-grade atrioventricular block and permanent pacemaker implantation (PPI), annular rupture, paravalvular leak (PVL), acute coronary obstruction, and access site-related vascular complications [5]. The incidence of new PPI after TAVI ranges from 10% to 20%, depending on the device type and patient population. This issue remains prevalent even with newer-generation valves and refined techniques [6]. Notably, TAVI patients who require a pacemaker have been shown to experience higher rates of morbidity and even increased mortality [7]. PVL, another well-recognized complication after TAVI, refers to regurgitant blood flow around the prosthetic valve due to incomplete sealing, and it was initially one of the most common TAVI-related complications [8]. Coronary artery obstruction is a rare but potentially catastrophic acute complication of TAVI. Occurring in roughly 0.5–1% of cases in native-valve TAVI, acute coronary obstruction often requires immediate percutaneous coronary intervention [9]. The mechanism typically involves displacement of the native aortic valve leaflets or calcific debris obstructing a coronary ostium during or after valve deployment. Access-site vascular complications represent another important category of acute TAVI complications, since it requires large-bore sheath insertion. Early-generation TAVI trials reported major vascular complications in up to 10–15% of cases, due to issues like arterial dissection, perforation, or access site bleeding [10]. With improved operator techniques, downsized delivery systems, and better closure devices, the incidence of major vascular events has declined substantially in recent years [10].

However, the complications rate in contemporary TAVI is still high and could impact long-term outcomes. This study aimed to assess the clinical and anatomical predictors of acute complications after TAVI with an emphasis on CT-derived anatomy of the aortic valve.

## 2. Materials and Methods

The research protocol complied with the Declaration of Helsinki and was approved by the local Ethics Committees of the Emergency Institute for Cardiovascular Diseases and Transplantation Târgu Mureș (approval number 2263/22.04.2025).

**Figure 1:**
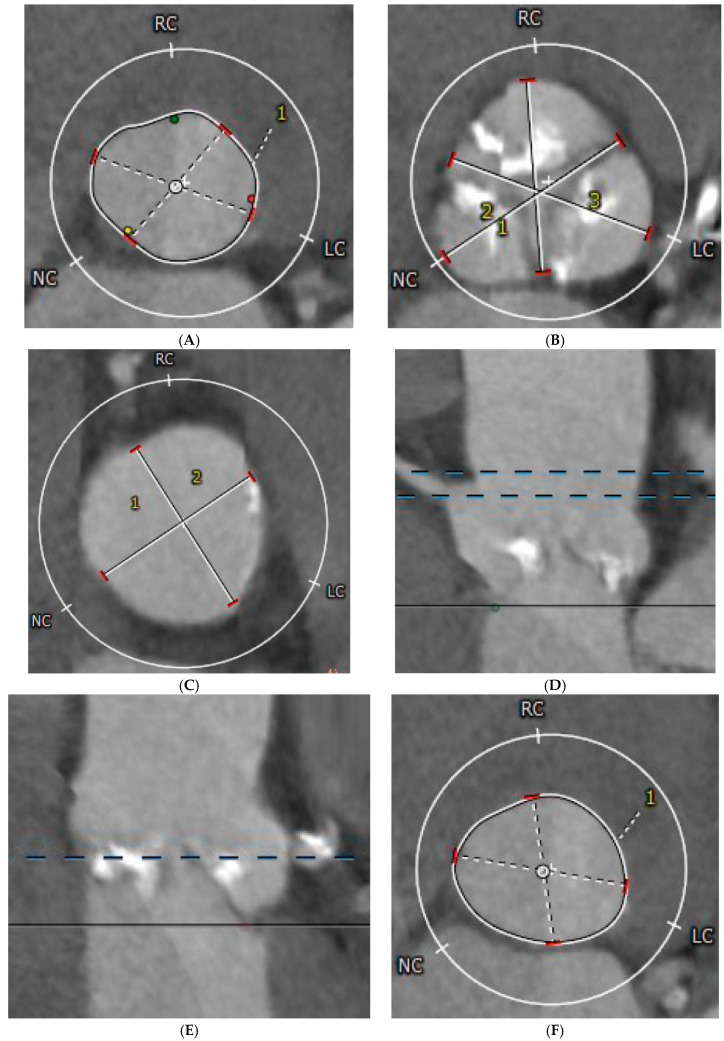
Computer-tomography-derived parameters of aortic valve anatomy. (**A**) Annulus perimeter, annulus area, minimum, and maximum annulus diameter; (**B**) Sinus of Valsalva diameter; (**C**) Sinotubular junction diameter; (**D**) Sinotubular junction and RCA height; (**E**) LCA height; (**F**) LVOT area. LC—left coronary cusp; LCA—left coronary artery; LVOT—left ventricular outflow tract; RC—right coronary cusp; NC—non-coronary cusp.

All patients who underwent the transfemoral TAVI procedure for severe degenerative aortic stenosis at our tertiary center were selected for inclusion in the present study. Inclusion criteria consisted of (1) TAVI performed during November 2016–May 2025 and (2) available CT-derived aortic valve anatomy. The main outcome of this study was the occurrence of any acute cardiac in-hospital complication. Exclusion criteria consisted of (1) age less than 18 years, (2) non-degenerative severe aortic stenosis (e.g., congenital), (3) TAVI intervention for aortic regurgitation, and (4) a permanent pacemaker present before the procedure, since it protects in case of de novo conduction disturbances. Femoral vascular complications were not considered in the present analysis, since they are related to the operator technique, and since all valve platforms have the same sheath diameter. The CT valve sizing was performed using 3mensio Structural Heart 10.1 SP3 software, and included anatomical parameters such as aortic annulus perimeter, area, minimum and maximum diameter, left ventricular outflow tract area, left coronary artery (LCA) and right coronary artery (RCA) height, sinus of Valsalva diameter, and sinotubular junction height and diameter (Figure 1). Data regarding anthropometrics, relevant medical history, clinical status at hospital admission, chronic medical treatment, routine laboratory parameters, echocardiographic parameters, and procedural characteristics were collected. The valve choice decision was at the discretion of the operator. Additionally, the pre- and post-dilatation decision was at the discretion of the operator on a case-by-case basis, based on the perceived valve expansion and stability, para- or intra-valvular leak, and intraoperative transprosthetic gradient.

The acute complications analyzed in the present study were defined according to the Valve Academic Research Consortium 3 and included (1) in-hospital death, (2) cardiac structural complications such as periprocedural acute myocardial infarction, acute coronary ostium closure requiring PCI, (3) perioperative AV block requiring permanent pacemaker implantation, (4) acute prosthesis dysfunction, (5) perioperative resuscitation or electrical cardioversion for malignant ventricular arrhythmias, and (6) perioperative cardiogenic shock with inotropic support, extracorporeal membrane oxygenation (ECMO), or intra-aortic balloon pump (IABP) support requirement [11]. Femoral vascular and access-related complications were not analyzed in the present study, as these are more closely related to the operative technique. Acute complications were considered those occurring during and after the TAVI procedure until the patient’s discharge.

A significance level α of 0.05 and a 95% confidence interval (CI) were considered. Continuous variables were evaluated for normal distribution using the Shapiro–Wilk test. Continuous variables with parametric distributions were reported as mean ± standard deviation and compared using the non-paired Student *t*-test, while continuous variables with non-parametric distributions and discrete variables were reported as median (interquartile range) and compared using the Mann–Whitney test. Categorical variables were reported as absolute and relative frequencies and compared using the Fisher exact test. The survival curves were assessed using the Kaplan–Meier method and compared using the log-rank test. To examine the impact of different predictors on survival, univariable and multivariable Cox proportional hazard models were used to predict the association in the form of a hazard ratio (HR) between observed survival and single or multiple independent variables, respectively. Multivariable models were constructed in a stepwise fashion, and overfitting was evaluated using Akaike’s Information Criterion (AIC) [12]. AIC was calculated at each step to assess if an overfitting problem was incurred by adding more variables to the model. Multicollinearity among independent variables was assessed, and a strong correlation between variables was considered present if the variance inflation factor was above 2.5. Missing values were handled using the population median imputation method [13]. Statistical analysis was performed using Python version 3.9.13.

## 3. Results

A total of 485 patients who underwent TAVI for severe degenerative aortic stenosis were included in this study. Baseline clinical and echocardiographic characteristics are summarized in Table 1. The median age of the cohort was 78 (75–82) years, and 276 patients (56.9%) were male. Balloon-expandable valves (BEVs) were implanted in 381 patients (78.5%), while self-expanding valves (SEVs) were used in 104 patients (21.4%). All valve implantations were performed using the standard three-cusp view.

A total of 69 (14.2%) patients suffered at least one acute complication following TAVI; in-hospital death occurred in nine patients, PPI was required in forty-four patients, intraoperative AV block occurred in eighteen patients, peri-operative resuscitation was performed in eighteen patients, electrical cardioversion was required in eight patients, peri-operative inotropic support was required in seven patients, ECMO/IABP was required in six patients, PCI for acute coronary ostium closure was required in four patients, and acute prosthesis dysfunction occurred in two patients. There was no significant difference in valve choice, pre- or post-dilatation rates among the operators, nor in the incidence of acute complications among operators.

Patients who experienced complications were significantly less likely to be male and had longer hospitalizations. Pre-existing left bundle branch block (LBBB) was more common among those who had complications, and patients with complications tended to have a larger left ventricular (LV) diameter. No significant differences were observed in any of the pre-procedural CT-derived aortic valve/anatomical measurements between patients with and without acute complications (Table 2). Main parameters of aortic root anatomy—including the annulus size (area, perimeter, minimal, and maximal diameters), sinus of Valsalva diameter, sinotubular junction dimensions, coronary artery heights, and LVOT area—were all comparable between the two groups.

**Figure 2:**
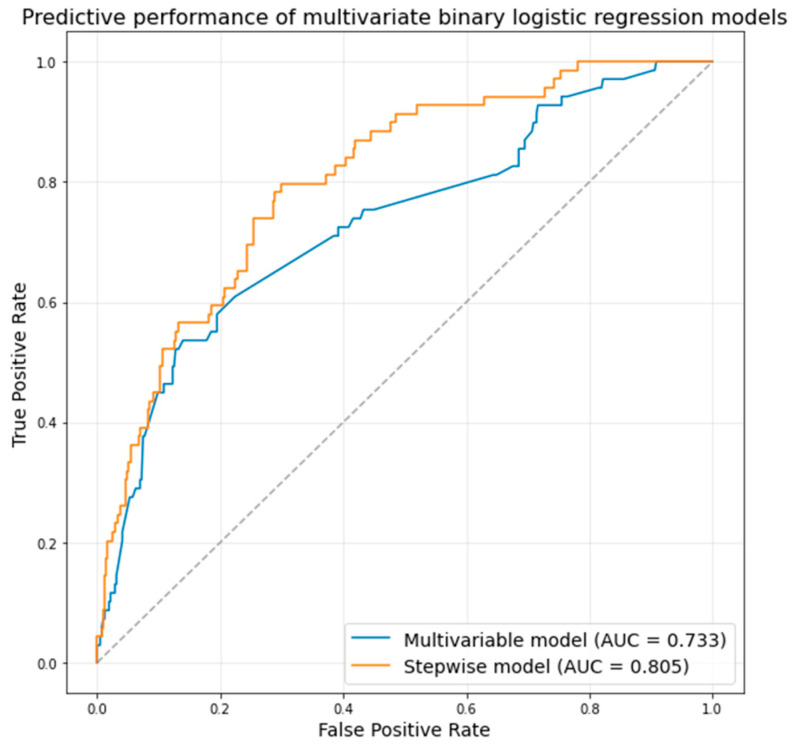
Predictive performance of the multivariable regression models. AUC—area under the receiver operator characteristic curve.

A significant association was found between the type of transcatheter valve used and the rate of acute complications (Table 3). Use of the Edwards Sapien 3 (balloon-expandable valve) was associated with a notably lower complication rate: 68.1% of patients in the complication group had a Sapien 3, compared to 80.3% of patients without complications (*p* = 0.02). Conversely, the self-expanding Navitor/Portico valve was more frequently implicated in complications: 13.0% of the complication group received a Navitor/Portico vs. only 4.1% of the no-complication group (*p* = 0.006). No other valve types showed a statistically significant difference in complication rates. In the multivariable logistic regression model (including only variables that were significant in univariate analysis), several factors were independent predictors of acute complications after TAVI (Table 4). The male sex remained significantly protective, with males having lower odds of complications (OR 0.45, 95% CI 0.25–0.81, *p* = 0.01). Pre-existing LBBB was confirmed as an independent risk factor (OR 2.19, 95% CI 1.06–4.49, *p* = 0.03), as was moderate-or-severe mitral regurgitation (OR 1.76, 95% CI 1.22–2.54, *p* = 0.002). A larger LV end-diastolic diameter showed a modest association with higher complication risk (OR 1.05 per mm increase, *p* = 0.04). The type of valve implant remained influential in the multivariable model: use of a Navitor/Portico valve was associated with over twice the odds of complications relative to other valves (OR 2.58, 95% CI 1.04–6.42, *p* = 0.04). Notably, an increased transprosthetic gradient after TAVI was linked to lower odds of complications (OR 0.91 per 1 mmHg increase in maximum gradient, *p* = 0.001). The area under the ROC curve of the multivariable model was 0.733 (Figure 2).

In the stepwise multivariable binary logistic regression model (Table 4), the male sex remained strongly protective (OR 0.31, *p* = 0.001), and both LBBB (OR 2.94, *p* = 0.01) and mitral regurgitation (OR 2.05, *p* = 0.004) continued to be significant risk factors. Moreover, diabetes mellitus emerged as an independent predictor in the stepwise model (OR 1.98, *p* = 0.03), as did a history of myocardial infarction (MI) (OR 2.96, *p* = 0.01), even though these factors did not show significant univariate differences between groups. The adverse impact of the Navitor/Portico valve was even more pronounced in the stepwise model (OR 4.84, *p* = 0.03). Finally, two anatomical parameters from CT gained significance in the stepwise analysis: a greater height of the left coronary artery (LCA) ostium was protective (OR 0.89 per mm, *p* = 0.03, indicating lower risk with higher LCA position), whereas a higher sinotubular junction (STJ) was associated with increased risk of complications (OR 1.16 per mm, *p* = 0.03). The predictive performance of the stepwise model was higher (Figure 2, area under ROC curve 0.805).

## 4. Discussion

The main results of this study can be summarized as follows: (1) a relatively high percentage of patients (14.2%) suffered at least one periprocedural complication, although in-hospital death was low (1.8%); (2) the Edwards Sapien 3 platform was associated with lower rates of acute complications; however, it was not significant in multivariable analysis. The Navitor/Portico platform was associated with higher rates of acute complications, which was also significant in multivariable analysis; (3) higher LCA height and lower sinotubular junction height were associated with lower rates of acute complications.

Our findings highlight the role of aortic root anatomy in TAVI-associated conduction disturbances. Consistent with other recent studies, specific annular and LVOT features measured on CT emerged as significant predictors of new PPI. A highly eccentric annulus (ellipticity ≥ 0.25) was associated with a markedly elevated PPI risk. Bianchini et al. reported that annular eccentricity ≥0.25 had an adjusted OR of 4.1 for PPI after TAVI with Sapien 3 valves [6]. Heavy calcification at the annulus (especially near the right coronary cusp) has also been implicated; high calcific volume at the RCC annulus nearly tripled PPI risk in that series [6]. These CT findings align with our observation that patients with asymmetric, calcified annuli are prone to periprocedural conduction block, likely due to compression or injury of the adjacent conduction system. Another key anatomical factor is the length of the membranous septum (MS) separating the aortic annulus from the His bundle. A short MS on pre-TAVI CT strongly predisposes to PPI, as the conduction system lies closer to the annulus. In the INTERSECT study, patients with new PPI had shorter MS lengths on average (3.7 mm vs. 4.1 mm) [14]. Notably, MS length was a significant predictor of PPI in both balloon-expandable and Evolut self-expanding valves, but not with the ACURATE neo self-expanding valves [14]. Hokken et al. further stratified MS length risk thresholds; an MS < 3 mm identified a high-risk phenotype with 30% PPI rate [15]. Those results underscore that annular geometry and septal anatomy on CT can be used to predict conduction outcomes, enabling personalized procedural strategies (e.g., alternative valve choice in high-risk anatomy). Baseline conduction disease remains an important modifier of these anatomic risks. Similar to prior reports, we found pre-existing left bundle branch block to be a powerful independent predictor of PPI [16]. Veulemans et al. observed an >11-fold higher odds of PPI with pre-TAVI right bundle branch block [16]. Other groups have noted that the pre-existing AV block or bifascicular block also heightens susceptibility to PPI [15]. Indeed, multivariable analyses show that a complex interaction of multiple factors, short MS, extensive annular calcification, deeper implant depth, and certain valve types (e.g., older self-expanding valves), all independently contribute to PPI risk.

Acute coronary obstruction is a rare (<1%) but often catastrophic complication of TAVI [17]. A low coronary ostium height and a small sinotubular junction or sinus of Valsalva size create a difficult anatomy for TAVI, where native leaflets or the prosthesis frame can block coronary flow [18]. Khan et al. analyzed multicentric registries and found optimal CT cut-offs for obstruction—coronary ostium height <11 mm (left main) or <15 mm (right coronary) and sinus of Valsalva width <30–31 mm were strongly predictive of coronary obstruction [18]. Additionally, a short vertical distance between the annulus plane and coronary ostia in combination with calcified leaflets can lead to ostial occlusion. In a study, native cusp height exceeding coronary height (e.g., the leaflet extends higher than the ostium) was 97% sensitive for predicting obstruction. Similarly, a virtually modelled valve-to-coronary distance <4 mm or leaflet calcium volume >600 mm^3^ had a 96% sensitivity [18]. These metrics essentially capture the same concept, insufficient sinus “clearance” for the displaced leaflets, and correlate with known anatomic risk factors (low coronaries, narrow sinuses). In our series, we routinely assessed coronary heights and sinus dimensions via CT; four cases presented acute left coronary artery closure that were successfully treated by PCI. Nonetheless, the COBRA study, for instance, showed in-hospital mortality of 27% once coronary obstruction occurs, underscoring why careful CT screening for these predictors is critical [9]. Our results are in line with the literature that CT planning is indispensable to identify features like low coronary height or small sinus size that would prompt protective strategies (e.g., protecting the left coronary artery with a guidewire during the procedure) to prevent this acute complication.

The type of transcatheter valve used has a known impact on certain acute outcomes, and our study’s results are in line with broader comparisons. We observed differences in complications between the balloon-expandable Sapien 3 (S3) and the self-expanding Portico/Navitor valves, which reflect known trade-offs reported by others. Generally, balloon-expandable valves (e.g., S3) tend to exert less radial force on the LVOT, translating to lower PPI rates, whereas self-expanding valves (first-generation CoreValve, Portico, etc.) often have more conformable frames that achieve larger effective orifice areas but at the cost of higher PPI and PVL in earlier iterations. Our data showed a higher PPI requirement with the Portico valve compared to S3, and this aligns with a recent propensity-matched analysis of 434 patients by Primessnig et al. In that study, the 30-day new PPI rate was 21.2% with Portico vs. 13.4% with Sapien 3 (*p* = 0.042) [19]. Likewise, the composite VARC-2 early safety endpoint was significantly worse in Portico recipients (9.2% vs. 3.7%, *p* = 0.03), driven in part by more frequent conduction issues. Our cohort, though smaller, demonstrated the same pattern, Portico valves had roughly double the pacemaker incidence of Sapien 3. In fact, implantation of a Portico valve itself was identified as an independent predictor of PPI in the Primessnig analysis (alongside factors like older age and pre-existing AV block). This suggests an intrinsic valve-related propensity, likely owing to Portico’s taller frame and need for deeper implantation in the LVOT. Conversely, Sapien 3′s supra-annular skirt and higher implant technique may protect the conduction system, resulting in consistently lower PPI rates across studies. The large OBSERVANT II registry in Italy corroborated this; at one year, Sapien 3 had the lowest permanent pacemaker rate (12.5%) compared to Evolut R/PRO, ACURATE neo, and Portico (15–22%, *p* < 0.01) [20]. Notably, Sapien 3′s PPI risk was about half that of Portico (12.5% vs. 22%) in that real-world series. Our findings add to this body of evidence, confirming that the choice of transcatheter valve influences acute conduction outcomes, with Sapien 3 outperforming early-generation self-expanders in this regard. The newer iterations of self-expanding valves have addressed prior shortcomings. In a recent head-to-head comparison, Navitor reduced paravalvular leak and major vascular complications compared with Portico [21]. However, the PPI rates between Navitor and Portico were not significantly different in published analyses (approximately 15–20% in both), indicating that the new sealing skirt did not worsen conduction issues [21]. Our Navitor data suggest that the incidence of new pacemakers with Navitor remained comparable to Portico’s, reflecting that while PVL and transfemoral profiles improved, conduction outcomes for this intra-annular valve still lag behind those of the Sapien 3. Lower PPI rates trend down with technical refinements. A recent study reported lower pacemaker rates with cusp-overlap implantation even for self-expanding valves [22].

Another procedural factor that we examined was the post-implant hemodynamic result, particularly the transprosthetic gradient. In our cohort, the vast majority of patients achieved low residual gradients (mean 8–10 mmHg), and we did not find a strong relationship between mildly elevated gradients and acute clinical events. This is in line with larger analyses suggesting that patient–prosthesis mismatch (PPM) after TAVI is usually mild and of limited immediate consequence. For instance, the SCOPE I trial noted Sapien 3 had higher gradients than a self-expander, yet no difference in 30-day or 1-year clinical outcomes. In OBSERVANT II, despite Sapien 3 having slightly higher discharge gradients, the 1-year composite outcomes were comparable among devices. Our data similarly suggest that an isolated moderate gradient (when the valve is functioning normally) does not typically cause acute instability. None of our patients with moderate PPM (indexed EOA 0.65–0.85 cm^2^/m^2^) had heart failure or needed intervention within 30 days. It appears that transprosthetic gradients in the single-digit to low-teens range are well-tolerated acutely, especially given TAVR’s tendency to avoid severe PPM in most cases (severe PPM incidence 1–3% in recent series). Indeed, TAVR generally confers larger effective orifice areas than surgical valves, particularly in small annuli, which has been a noted advantage. That said, we acknowledge that any significant residual gradient should be avoided, when possible, as it represents suboptimal leaflet expansion or undersizing. In one patient with a small annulus in our study, we performed additional post-dilation upon measuring a mean gradient of 20 mmHg intra-procedurally, successfully dropping it to 10 mmHg and preventing early prosthesis–patient mismatch.

The present study has several strengths and limitations. The main limitation of this study is its retrospective nature. Furthermore, our study is a non-randomized observational study, which inherently carries a risk of residual confounding. We employed multivariable adjustments to account for baseline differences, but unmeasured factors could have influenced outcomes; thus, our findings would require external validation. However, this study included a relatively large number of valve platforms, especially for the SEV. Another important limitation of our study is that certain important parameters (e.g., calcium score) were not available, and other TAVI approaches were employed (e.g., cusp-overlap technique).

## 5. Conclusions

A high acute complications rate is typical for TAVI even though most complications can be successfully treated and the in-hospital death rate is low. Clinical parameters—such as diabetes mellitus, LBBB, or LV diameter—and anatomic parameters such as LCA height and sinotubular junction height, were predictors of complications. Periprocedural characteristics, such as maximum transprosthetic gradient and the use of the Portico/Navitor valve platform were also associated with the occurrence of acute complications.

## Figures and Tables

**Table 1:** Clinical characteristics of the studied population.

Parameter	All Patients (*n* = 485)	Acute Complications		
		Without (*n* = 416)	With (*n* = 69)	*p*-Value
Age (years)	78 (75–82)	78 (75–82)	76.94 ± 6.87	0.23
Male sex	276 (56.9%)	245 (58.9%)	31 (44.9%)	0.03
Hospitalization (days)	6.8 (4.9–8.8)	6.2 (4.9–7.9)	10.9 (7.0–14)	0.001
Diabetes mellitus	177 (36.5%)	148 (35.6%)	29 (42.0%)	0.34
Hypertension	415 (85.6%)	360 (86.5%)	55 (79.7%)	0.14
Atrial fibrillation	179 (36.9%)	157 (37.7%)	22 (31.9%)	0.41
Chronic kidney disease	80 (16.5%)	71 (17.1%)	9 (13.0%)	0.48
Stroke	30 (6.2%)	26 (6.2%)	4 (5.8%)	1.00
Prior MI	49 (10.1%)	38 (9.1%)	11 (15.9%)	0.08
Prior CABG	19 (3.9%)	17 (4.1%)	2 (2.9%)	1.00
LBBB	62 (12.8%)	48 (11.5%)	14 (20.3%)	0.05
Active smoker	12 (2.5%)	11 (2.6%)	1 (1.4%)	1.00
Dyslipidemia	235 (48.5%)	211 (50.7%)	24 (34.8%)	0.01
COPD	34 (7.0%)	31 (7.5%)	3 (4.3%)	0.45
CAD	86 (17.7%)	80 (19.2%)	6 (8.7%)	0.03
DCM	32 (6.6%)	29 (7.0%)	3 (4.3%)	0.60
Creatinine (mg/dl)	1.08 (0.86–1.31)	1.08 (0.87–1.33)	1.06 (0.84–1.27)	0.44
Hemoglobin (g/dL)	12.9 (11.7–14)	12.8 ± 1.72	12.5 ± 1.73	0.16
Leucocytes (×10^3^/µL)	7.04 (5.92–8.45)	7.11 (5.96–8.56)	6.65 (5.85–7.86)	0.06
Platelets (×10^3^/µL)	203 (169–247)	203 (170–247)	200 (164–244)	0.46
LVEF (%)	50 (45–55)	50 (45–55)	50 (45–55)	0.42
LV diameter (mm)	51 (45–56)	50.50 (45–56)	53.79 ± 8.70	0.05
Maximum gradient (mmHg)	79 (65–94)	79 (66–93)	78 (60–96)	0.63
Mean gradient (mmHg)	47 (40–59)	47 (40–59)	50.5 ± 21.8	0.77
AVA (cm^2^)	0.66 ± 0.16	0.65 ± 0.17	0.68 ± 0.10	0.73
Maximum transprosthetic gradient (mmHg)	20 (13–26)	21 (14–28)	14.94 ± 6.84	0.001
Mean transprosthetic gradient (mmHg)	11 (8–15)	11 (8–15)	8.42 ± 3.56	0.008
Valve implantation depth (mm)	3.9 (3.5–4.5)	3.8 (3.5–4.4)	4.1 (3.4–4.8)	0.12
EuroSCORE I (%)	8.1 (5.7–13.8)	8.1 (5.6–13.3)	8.6 (6.1–14.6)	0.41
EuroSCORE II (%)	4.6 (2.3–15.1)	4.2 (2.3–13.9)	11.0 (3.1–19.7)	0.39

AVA—aortic valve area; CABG—coronary artery bypass graft; CAD—coronary artery disease; COPD—chronic obstructive pulmonary disease; DCM—dilated cardiomyopathy; HBP—high blood pressure; LBBB—left bundle branch block; LV—left ventricle; LVEF—LV ejection fraction; MI—myocardial infarction.

**Table 2:** CT-derived aortic valve anatomic parameters.

Parameter	All Patients (*n* = 485)	Acute Complications		
		Without (*n* = 416)	With (*n* = 69)	*p*-Value
Annulus area (mm^2^)	463 (410–528)	463 (409–527)	472 ± 92	0.98
Annulus perimeter (mm)	77 (72–83)	78 (72–83)	78.24 ± 7.63	0.98
Minimum annulus diameter (mm)	21.4 (20.0–23.1)	21.4 (20.0–23.1)	21.61 ± 2.45	0.96
Maximum annulus diameter (mm)	27 (25–29)	27 (25–29)	27.7 ± 2.5	0.75
Sinus of Valsalva diameter (mm)	30.61 ± 3.55	30.63 ± 3.51	30.48 ± 3.89	0.79
Sinotubular junction diameter(mm)	28.6 (25.9–30.9)	28.6 (26–31)	28 (25–30)	0.35
LCA height (mm)	14 (12–16)	14 (12–16.32)	12.0 (11–15)	0.06
RCA height (mm)	17.9 (15.5–20)	17.9 (15.5–20)	17 (15–19)	0.36
Sinotubular junction height (mm)	23.4 (21.5–25.5)	23.4 (21.5–25.6)	26.4 (22.5–28.0)	0.07
LVOT area (mm)	462 (406–530)	464 (404–530)	475 ± 89	0.90
Annulus ellipticity (%)	22 (17–26)	22 (17–26)	22.06 ± 6.35	0.70
LVOT ellipticity (%)	24.11 ± 6.89	24.10 ± 6.76	24.21 ± 7.77	0.92

LCA—left coronary artery; LVOT– left ventricular outflow tract; RCA—right coronary artery.

**Table 3:** Association between valve type and complication occurrence.

Valve Type	Acute Complications		*p*-Value *	OR (95% CI)	*p*-Value **
	Without (*n* = 416)	With (*n* = 69)			
EdwardsSapien3	334 (80.3%)	47 (68.1%)	0.02	0.52 (0.29–0.91)	0.02
Navitor/Portico	17 (4.1%)	9 (13.0%)	0.006	3.52 (1.52–8.29)	0.003
Medtronic	11 (2.6%)	3 (4.3%)	0.43	1.67 (0.45–6.15)	0.43
Accurate	28 (6.7%)	4 (5.8%)	1.00	0.85 (0.28–2.51)	0.77
Boston	26 (6.2%)	6 (8.7%)	0.43	1.42 (0.56–3.60)	0.45

OR—odds ratio. * *p*-value was obtained using the Fisher test. ** *p*-value was obtained using univariable logistic regression.

**Table 4:** Multivariable prediction of acute complications occurrence after TAVI.

Parameter	Multivariable Regression *		Stepwise Regression **	
	OR	*p*-Value	OR (95% CI)	*p*-Value
Male sex	0.45 (0.25–0.81)	0.01	0.31 (0.15–0.63)	0.001
Diabetes mellitus	-	-	1.98 (1.08–3.64)	0.03
History of MI	-	-	2.96 (1.24–7.04)	0.01
LBBB	2.19 (1.06–4.49)	0.03	2.94 (1.37–6.31)	0.01
LV diameter	1.05 (1.00–1.09)	0.04	-	-
Mitral regurgitation	1.76 (1.22–2.54)	0.002	2.05 (1.38–3.04)	0.004
Transprosthetic gradient	0.91 (0.86–0.97)	0.001	0.90 (0.84–0.96)	0.008
Navitor/Portico valve	2.58 (1.04–6.42)	0.04	4.84 (1.13–20.71)	0.03
LCA height	-	-	0.89 (0.8–0.99)	0.03
Sinotubular junction height	-	-	1.16 (1.01–1.32)	0.03

LBBB—left bundle branch block; LCA—left coronary artery; LV—left ventricle; MI—myocardial infarction; OR—odds ratio. * The multivariable model was built using significant variables in univariate analysis. ** The multivariable model was built using stepwise AIC.

## Data Availability

The raw data supporting the conclusions of this article will be made available by the authors on request.
